# Case report: Systemic multi-organ involvement in an adult-onset immunodeficiency patient infected with *Talaromyces marneffei*

**DOI:** 10.3389/fimmu.2024.1430179

**Published:** 2024-09-09

**Authors:** Kun Li, Yuping Zhang, Dan Zhang, Qing Chen, Xueling Fang

**Affiliations:** ^1^ Department of Critical Care Medicine, The First Affiliated Hospital, Zhejiang University School of Medicine, Hangzhou, Zhejiang, China; ^2^ Department of Laboratory Medicine, The First Affiliated Hospital, Zhejiang University School of Medicine, Hangzhou, Zhejiang, China; ^3^ Department of Pathology, The First Affiliated Hospital, Zhejiang University School of Medicine, Hangzhou, Zhejiang, China

**Keywords:** Talaromyces marneffei, adult-onset immunodeficiency, respiratory failure, immunodeficiency, interferon-γ

## Abstract

Adult-onset immunodeficiency (AOID) mediated by anti-interferon-γ autoantibodies (AIGA) is a rare condition, particularly prevalent in Southeast Asia and southern China. We present a case study of a 62-year-old female with AOID who developed a severe pulmonary infection caused by *Talaromyces marneffei* (TM), leading to acute respiratory failure, generalized rash, multiple lymphadenopathies, bone destruction, and a mediastinal mass. Treatment included mechanical ventilation, antifungal medication, and corticosteroids, resulting in complete recovery and discharge. This case underscores the challenges of managing complex infections in AOID patients and highlights the importance of early diagnosis through metagenomic next-generation sequencing (mNGS) and appropriate intervention to improve clinical outcomes.

## 1 Introduction

Adult-onset immunodeficiency (AOID) is a rare condition mediated by autoantibodies against interferon-γ (anti-interferon-γ autoantibodies, AIGA). Initially, AIGAs were considered a sporadic late-onset immunodeficiency observed in patients with mycobacterial infections (1). It is more prevalent in populations from Southeast Asia and southern China, with an estimated incidence rate of 0.5-1.0 per million people (1–4). The earliest documented cases internationally were reported around the year 2004 (5–7). Since 2012, several cohorts of patients with AIGAs have been reported in Thailand, Taiwan, Japan, and South China. Additionally, sporadic cases have been documented throughout Southeast Asia, including the Philippines, Vietnam, Cambodia, Singapore, and Malaysia (2, 8–10). To date, over 600 patients with AIGAs have been diagnosed, predominantly among adults of Asian descent, with more than two-thirds of the cases reported in Thailand and Taiwan (2–4, 8–12). Currently, reported cases of AOID in China are primarily concentrated in southern provinces such as Guangxi, Guangdong, and Taiwan (3, 4, 8, 10, 13–16). AIGAs have been detected in patients across a wide age range, from 10 to 87 years, with most cases occurring in adults around 50 years old (2, 3, 9, 10, 12). Although autoimmune diseases generally display a female preponderance, there is no obvious sex bias in patients with AIGAs (male/female ratio = 0.82) (2, 3, 9, 10, 12).

The initial clinical signs and symptoms of AIGA-related disease predominantly result from disseminated non-tuberculous mycobacterial (NTM) infections (8, 9). These often include bilateral cervical or generalized lymphadenitis and associated reactive skin lesions. Additionally, non-specific symptoms such as fever, malaise, cough, bone pain, and weight loss may be observed, similar to those seen in various other diseases (8–10). Mycobacteria are the most frequently observed pathogens, affecting 85.5% of patients with AIGAs and often causing severe, disseminated infections (2, 3, 9). Invasive non-typhoid salmonellosis is the most common bacterial infection observed in patients with AIGAs. Cohort studies have reported that it affects 29–40% of these patients (2, 3, 12). Shingles, caused by the varicella–zoster virus (VZV), is the most common viral infection observed in patients with AIGAs (2, 3, 12). A cohort study reported that up to 62.2% of these patients had a history of shingles, which is notably higher than the 32% lifetime risk observed in the general population (3). Additionally, VZV, herpes simplex virus (HSV), and cytomegalovirus (CMV) infections have been documented in patients with Mendelian susceptibility to mycobacterial disease (MSMD), particularly those with interferon-γ (IFN-γ) receptor 1 (IFN-γR1) deficiency (17). Fungal infections, such as cryptococcosis, aspergillosis, and candidiasis, have been observed in patients with AIGAs (2, 3, 12, 18). Moreover, AIGAs have been shown to be associated with severe *Talaromyces marneffei* (TM) infections in human immunodeficiency virus (HIV)-negative patients (10). TM is the second most common pathogen in these cases, likely due to its high prevalence in Southeast Asia (19). Additionally, AIGA can lead to severe opportunistic infections that are difficult to control, particularly in patients with recurrent infections (19). Therefore, this condition requires significant clinical attention.

TM is a significant pathogenic thermally dimorphic fungus that causes systemic mycosis in Southeast Asia (20–22). In 1956, TM was initially isolated from the liver of a bamboo rat (23, 24). The first documented natural human infection was reported in 1973, involving an American minister with Hodgkin’s disease who lived in Southeast Asia (25). Historically, TM infection in humans has been considered exclusively associated with acquired immunodeficiency syndrome (AIDS) caused by HIV infection (20, 26). However, in recent years, there have been increasing reports of *Talaromyces* infections in non-HIV-infected individuals (22). Most of these cases involve other immunosuppressive conditions, including primary immunodeficiencies, autoimmune diseases, malignancies, and iatrogenic immunosuppression (27). Currently, bamboo rats and various other rodents are frequently identified as reservoirs for TM infections (20, 28). TM has the ability to enter the body’s reticuloendothelial system, travel through the bloodstream, and infect other organs. This can occur through the respiratory and digestive tracts, potentially leading to severe systemic infections and presenting life-threatening risks, especially in individuals with weakened immune systems (27, 29).

To improve empirical treatment decisions and enhance patient survival, it is crucial to rapidly identify pathogens in patients infected with TM. Metagenomic next-generation sequencing (mNGS) tests, an advanced nucleic acid detection technology for pathogen identification, have the capability to detect a broad spectrum of pathogens, predict antibiotic resistance, and deliver results within 24 hours (30, 31). Cohort studies focused on respiratory tract infections have demonstrated that the positive rate of mNGS (>60%) for detecting respiratory pathogens is significantly higher than that of traditional microbial detection methods (30–50%) (32, 33). Notably, mNGS has a significant advantage in diagnosing unexplained infections and co-infections (34).

Cases of AOID infection with TM are relatively rare. We report a case of a diagnosed AOID patient who was infected with TM identified through mNGS, presenting with severe pulmonary infection, acute respiratory failure, generalized rash, multiple lymphadenopathies, bone destruction, and a mediastinal mass. The patient was successfully treated with mechanical ventilation, antifungal medication, management of shock, and corticosteroids, leading to complete recovery and discharge from the hospital.

## 2 Case presentation

A 62-year-old female patient, a farmer by trade, with no history of chronic diseases such as diabetes, no infectious disease history, no history of trauma or surgery, and no significant family history, was admitted to the Oncology Department of the First Affiliated Hospital of Zhejiang University School of Medicine on January 5, 2024, due to “headache and neck pain for 2 weeks.”

Two weeks before admission, the patient developed headaches accompanied by neck pain, numbness in both hands, and fever (highest temperature approximately 37.6°C), and sought medical attention at a local hospital (First People’s Hospital of Lin’an District, Hangzhou). Blood tests showed a white blood cell count (WBC) of 19.5 x10E9/L, C-reactive protein (CRP) of 223.1mg/L, total protein of 90.3 g/L, globulin of 54.8 g/L, and an erythrocyte sedimentation rate (ESR) of 120 mm/h. Chest CT revealed irregular mass in the left upper mediastinum, soft tissue nodules and masses adjacent to the left side of the aortic arch in the mediastinum, suggestive of a tumorous lesion, with a C6 spinous process fracture.

On December 29, 2023, a biopsy of the mass above the left clavicle was performed, showing lymphocyte proliferation in fibrous tissue, predominantly T lymphocytes, suggesting the need for further lymph node excision to exclude vascular immunoblastic lymphoma. Lumbar puncture and routine cerebrospinal fluid analysis and general bacterial culture were unremarkable. The local hospital diagnosed a mediastinal mass, lymph node enlargement, and cervical spine fracture, and treated the patient with piperacillin-tazobactam for infection, celecoxib for fever and pain relief, among other treatments. However, the patient’s condition worsened, leading to transfer to our hospital and admission to the Oncology Department.

**Figure 1:**
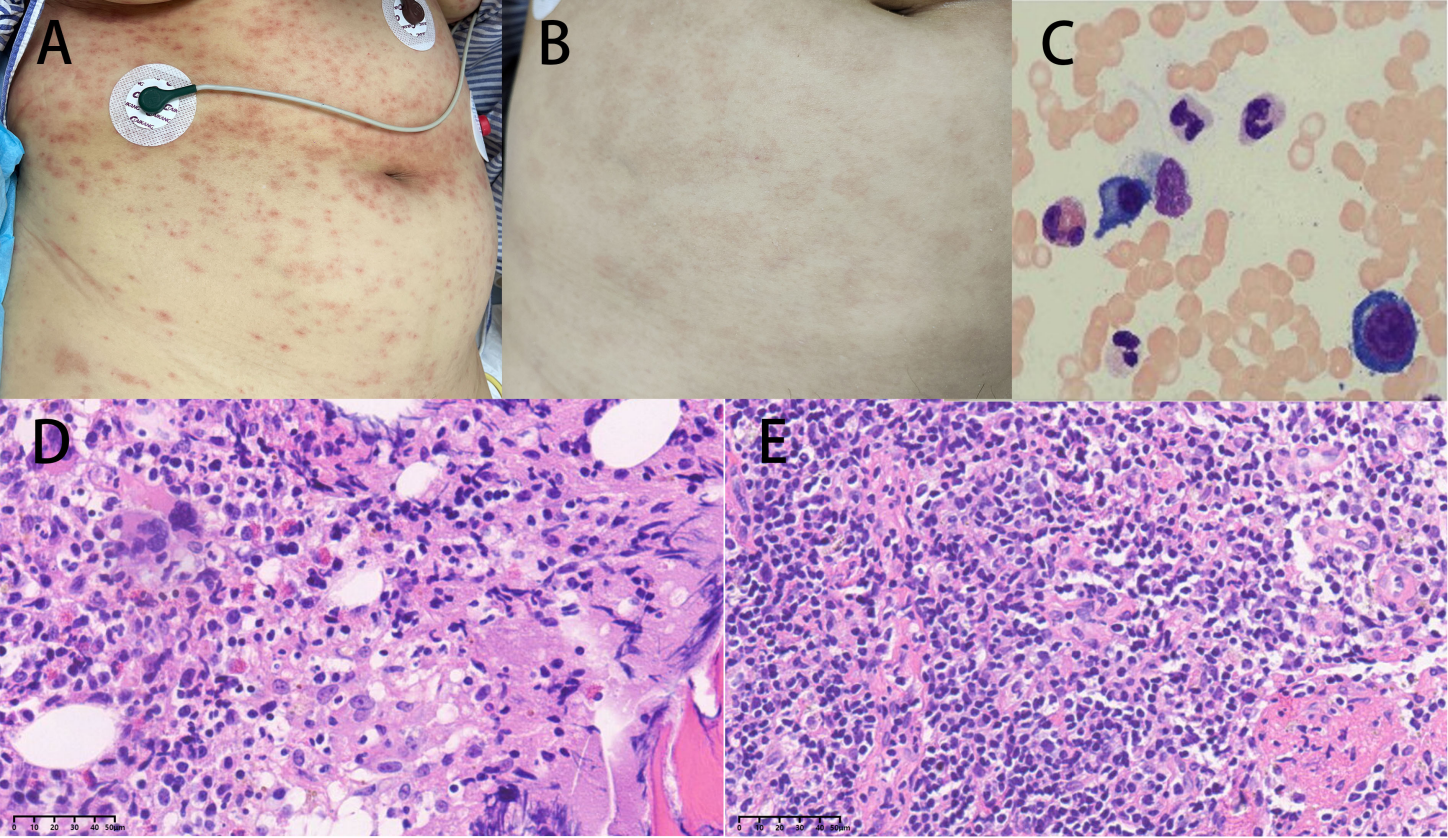
**(A)** Upon admission, the patient had scattered reddish-brown papules on the whole body. **(B)** After treatment, the rash significantly subsided. **(C)** The bone marrow showed an increased proportion of plasma cells. **(D)** The hematopoietic tissue in the bone marrow was highly active (70-80% cellularity), accompanied by active proliferation of T lymphoid tissue and increased plasma cells (magnification × 40). **(E)** (Histopathology from lymph node puncture above the left clavicle) showed active proliferation of lymphoid tissue with a predominance of neutrophils, plasma cell infiltration, and tendencies toward inflammatory changes (magnification × 40).

Upon admission, the patient’s physical examination revealed a body temperature of 36.5°C, a pulse rate of 104 beats per minute, a respiratory rate of 19 breaths per minute, and a blood pressure of 153/84 mmHg, with a pain score (visual analogue scale, VAS) of 1 point. The patient was alert and oriented, and the neurological examination showed no abnormalities. Scattered reddish-brown maculopapular lesions were observed throughout the body, with a higher concentration on the trunk, some exhibiting a ring-shaped appearance but without significant infiltration (**Figure 1A**). A palpable mass, approximately 3 × 4 cm in size, with a tough texture and limited mobility, was detected above the left collarbone, with no tenderness noted. Additionally, coarse breath sounds were heard in both lungs, with no noticeable crackles or wheezes.

Routine laboratory tests showed the following results: white blood cell count (WBC) 21.04 × 10^9^/L, neutrophils 19.36 × 10^9^/L, lymphocytes 1.03 × 10^9^/L, hemoglobin 100 g/L, red blood cell count 4.05 × 10^12^/L, platelet count 192 × 10^9^/L, B-type natriuretic peptide precursor quantification 1171 pg/mL, activated partial thromboplastin time 46.0 seconds, prothrombin time 14.0 seconds, D-dimer 6830 μg/L, creatinine 138 μmol/L, total bilirubin (TBIL) 33.5 μmol/L, direct bilirubin (DBIL) 26.2 μmol/L, globulin 55.2 g/L, albumin 23.1 g/L, C-reactive protein (CRP) 351.30 mg/L, procalcitonin 0.75 ng/mL, glycated hemoglobin A1c 6.7%, immunoglobulin G 2450.0 mg/dL, cancer antigen 125 101.9 U/mL, ferritin 2331.4 ng/mL. Serum protein electrophoresis (M protein) showed: albumin 34.2%, α1 6.3%, β 8.2%, γ 40.3%. Thyroid function tests revealed: tetraiodothyronine 47.44 nmol/L, triiodothyronine 0.31 nmol/L, thyroid-stimulating hormone 0.218 mIU/L, free triiodothyronine 1.96 pmol/L. Immunoglobulin free light chain assay showed: kappa free light chain 89.0 mg/L, lambda free light chain 111.0 mg/L, free light chain ratio 0.80. HIV negative. Epstein-Barr virus antibody (IgM) negative. The complete laboratory results throughout the course of the illness are summarized in **Table 1**.

**Table 1:** Biological parameters of the patient.

	Reference	Jan 5	Jan 9	Jan 10	Jan 11	Jan 12	Jan 14	Jan 15	Jan 18	Jan 19	Jan 22
WBC (10^9^/L)	4-10	21.04	17.89	14.44	14.93	13.99	16.60	12.53	16.70	11.52	9.15
Neutrophil(10^9^/L)	2-7	19.36	16.35	13.79	13.94	12.26	15.37	11.44	16.05	10.73	8.07
Lymphocytes (10^9^/L)	0.8-4.0	1.03	0.63	0.40	0.70	1.51	1.03	0.91	0.27	0.50	0.57
HB (g/L)	113-151	100.00	84.00	79.00	76.00	73	68	65	65	56	62
PLT (10^9^/L)	101-320	192.00	70.00	38.00	31	33	48	69	161	143	206
G testing (pg/ml)	1-60				80.80				<10.00		<10.00
IgA (mg/dL)	100.0-420.0		309.00					236			178
IgM (mg/dL)	50.0-280.0		96.90					80.4			69.7
IgG (mg/dL)	860.0-1740.0		2450.00					1510			1260
HIV (S/CO)		negative									
IFN-α (pg/mL)	0-8.50				0.10						5.56
IFN-γ (pg/mL)	0-7.42				10.21						10.27
CRP (mg/L)	0-8	182.50	351.30	337.80	178.70	85.80	23.80	14.30	12.70	33.90	60.2
PCT (ng/mL)	0-0.5	0.75	2.52	2.13	1.98	1.56	0.64	0.46	0.25	0.29	0.32
T. BIL (umol/L)	0.0-21.0	11.60	33.50	58.00	50.30	39.5	55.5	52.1	65.7	45.9	27.6
D. BIL (umol/L)	0.0-8.0	7.90	26.20	49.70	43.20	31.6	45.5	43.2	52.2	33.6	18.7
ALT (U/L)	7-40	16.00	7.00	8.00	11.00	16	28	26	35	32	31
AST (U/L)	13-35	14.00	12.00	14.00	16.00	33	39	28	36	26	25
GGT (U/L)	7-45	140.00	62.00	40.00	32.00	32	75	85	185	171	177
LDH (U/L)	120-250		164.00	235.00	279.00	373	272	242	248	207	196
AKP (U/L)	50.0-135.0	320.00	184.00	150.00	120.00	106	81	74	144	153	214
ALB (g/L)	40.0-55.0	23.10	23.00	24.20	23.80	24.1	27.7	27.1	33.1	32.1	32.7
Globulin (g/L)	20.0-40.0	55.20	40.80	39.40	36.30	35.1	28	27	23.6	21.5	24.9
GLU (mmol/L)	3.90-6.10			8.50	10.00	12.10	9.6	11.20	10.60	8.70	8.2
BUN (mmol/L)	3.00-8.80	5.50	4.30	7.30	9.60	15.30	13.10	11.50	11.40	8.70	8.3
Cr (µmol/L)	41-81	47.00	39.00	41.00	52.00	63	56	57	50	48	38
GFR (EPI-cr)		101.30	107.70	105.90	98.00	90.5	95.6	95	99.2	100.6	108.6
PT (S)	10-13.5	14.00	15.20	15.80	14.30	14.6	14.7	15.7	12.4	12.8	12.3
APTT (S)	23.9-33.5	26.90	34.70	32.70	30.10	28.7	29.4	29.4	22.7	23.8	27.2
D-dimer(ug/L FEU)	0-700	6830.00	8200.00	7440.00	3540.00	4230	5730	7940	7090	7590	5360
HbAlc (%)					6.70						
OI (mmHg)			192.00	91.40	214.50	229.75	265	249.25	374.29	305	386.67
LAC(mmol/L)	0.5-2.2			2.60	3.50	1.8-2.6	1.90	1.50	2.10	1.90	1.7
T (°C)		36.7	37.4	37.1	37.7	37.90	37.30	36.50	36.70	37.00	37.30
CD4^+^/CD8^+^	0.71-2.78										1.02
CD19+	90-560										27
CD3+	955-2860										413
TB						negative	negative	negative			

WBC, white blood cell; HB, hemoglobin; PLT, platelet; CRP, C-reactive protein; PCT, procalcitonin; T. BIL, total bilirubin; D. BIL, direct bilirubin; TBA, total bile acid; AST, aspartate aminotransferase; ALT, alanine aminotransferase; LDH, lactate dehydrogenase; GGT, gammaglutamyl transpeptidase; AKP, alkaline phosphatase; GLU, Serum glucose; LPS, lipase; Cr, creatinine; PT, prothrombin time; APTT, activated partial thromboplastin time; HbA1c,Serum Glycated hemoglobin A1c; G testing, (1,3)-β-D-glucan Testing; OI, Oxygenation Index; T, body temperature; LAC, Whole blood lactic acid; D-dimer, Plasma D-dimer; β-hydroxybutyric acid, serum β-hydroxybutyric acid; HIV, human immunodeficiency virus antigen-antibody; GFR, glomerular filtration rate. IgA, immunoglobulin A; IgM, immunoglobulin M; IgG, immunoglobulin G; CD4^+^/CD8^+^, ratio of helper/suppressor T lymphocytes (CD4^+^/CD8^+^); CD19^+^, absolute count of B lymphocytes (CD19^+^); CD3^+^, absolute count of T lymphocytes (CD3^+^); TB, tubercle bacillus smear examination.

**Figure 2:**
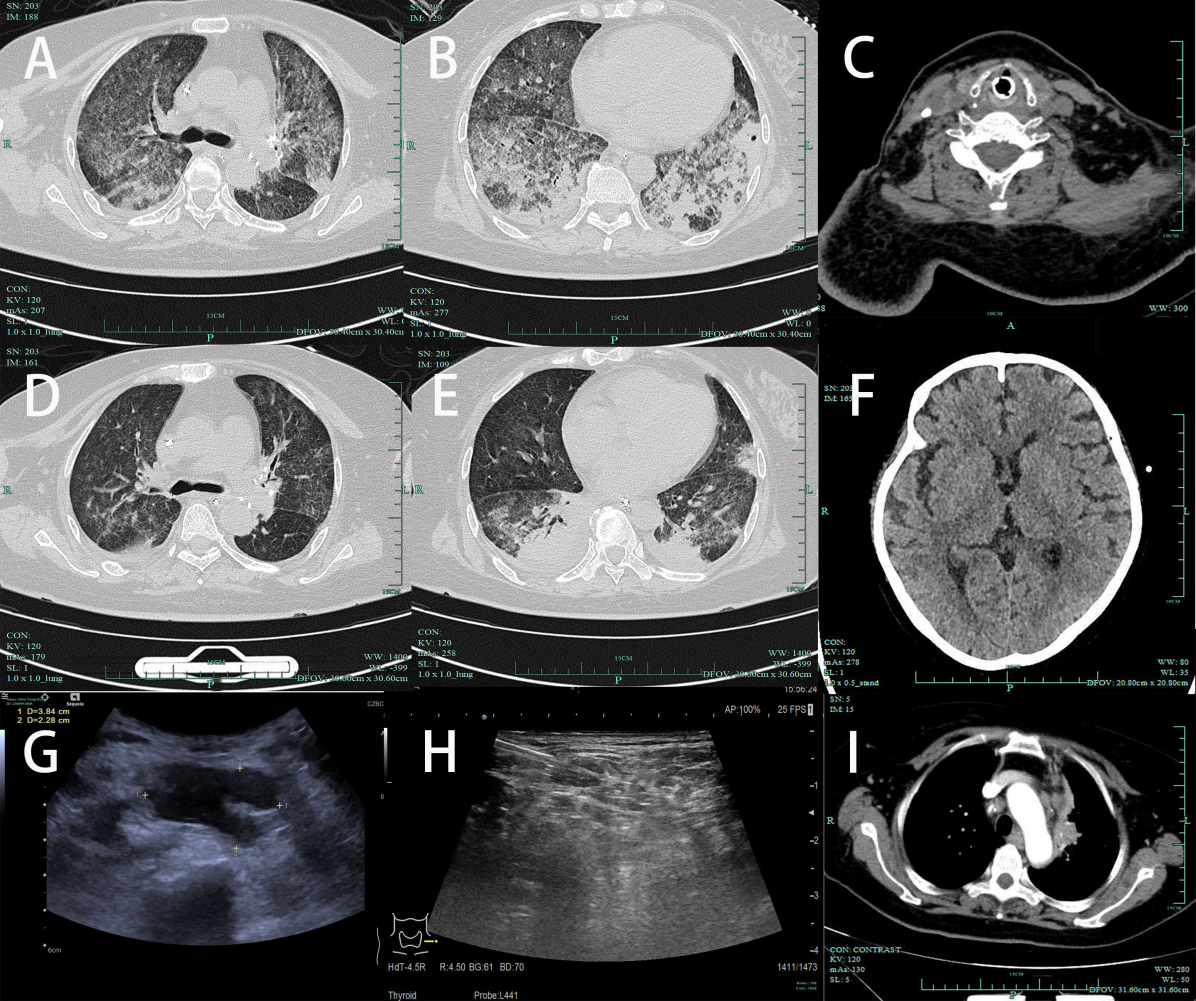
**(A, B)** Chest CT (2024-01-11): Diffuse infiltration with consolidation in both lungs, accompanied by local cavitation. **(C)** Neck CT (2024-01-11): Destruction of the C6 vertebral body and spinous process with soft tissue mass formation. **(D, E)** After treatment, follow-up chest CT on 2024-01-16 showed significant absorption of lung patchy shadows compared to before, with a reduction in local cavities. **(F)** Head CT (2024-01-16): Localized low-density shadow in the frontal bone, suggesting bone destruction. **(G)** Neck ultrasound: Bilateral supraclavicular multiple lymph node enlargement, hypoechoic mass in the left supraclavicular region, measuring 3.84cm × 2.28cm; **(H)** Post-treatment on 2024.1.16, reduction in lymph nodes in the supraclavicular region. **(I)** On admission, enhanced CT examination of the chest revealed an irregular mass in the left upper mediastinum.

The special examination results revealed several significant findings. Neck ultrasound showed bilateral supraclavicular lymphadenopathy and a hypoechoic mass in the left supraclavicular region (**Figure 2G**). Chest CT scan displayed diffuse infiltrates with consolidation in both lungs, along with local cavitation (**Figures 2A, B**). Enhanced CT examination of the chest revealed an irregular mass in the left upper mediastinum (**Figure 2I**). Neck CT scan indicated bone structure destruction at various sites, including the slope, left occipital condyle, C6 vertebral body, and spinous process, accompanied by soft tissue mass formation (**Figure 2C**). Head CT scan showed localized low-density shadows in the frontal bone and soft tissue density shadows with bone destruction at the slope (**Figure 2F**). Bone marrow cytology indicated an increased ratio of plasma cells (**Figure 1C**), while histology showed highly active hematopoietic tissue with active proliferation of T lymphoid tissue and increased plasma cells (**Figure 1D**). Pathological examination of a lymph node biopsy from the left supraclavicular region revealed minimal lymphoid tissue proliferation with specific immunohistochemical staining patterns (**Figure 1E**). Additionally, ultrasound-guided aspiration of a left neck mass yielded purulent fluid for microbial culture.

After admission, the patient received treatment including levofloxacin for infection, acetaminophen for fever reduction, sustained-release tramadol for pain relief, and cervical collar immobilization.

**Figure 3:**
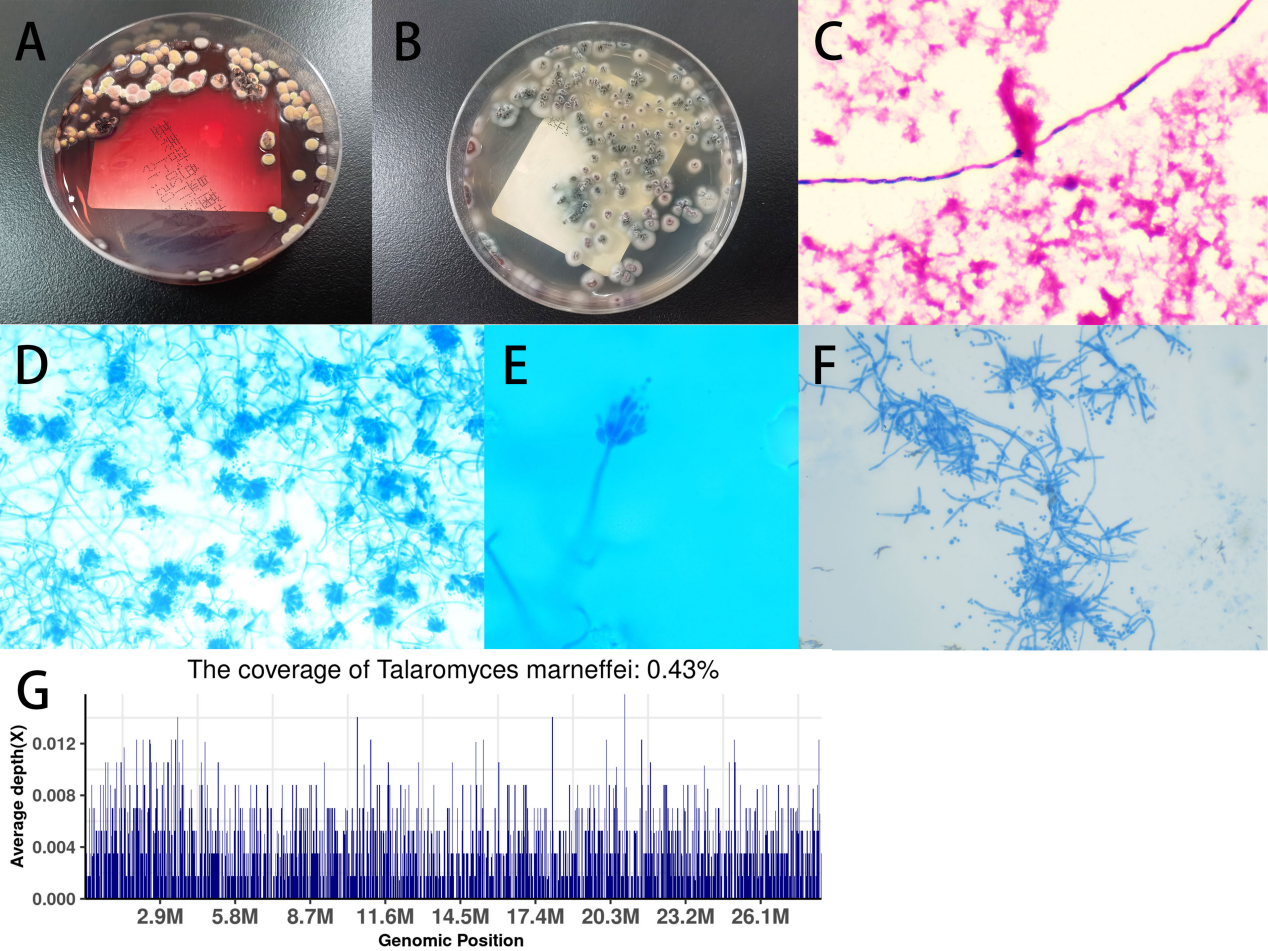
**(A)** Blood culture showed *Talaromyces marneffei* (TM) in the mold phase at 25°C, with characteristic red pigment production observed; **(B)** Culture of pus sample from left neck mass on Sabouraud’s agar medium plate at 37°C showed yeast phase; **(C)** Blood culture sample showed TM under microscopy (magnification × 400); **(D)** Microscopic analysis of colonies grown on Sabouraud’s agar medium plate at 25°C using lactophenol cotton blue staining revealed scattered, slightly asymmetric, typical double-helical broom-like fungal hyphae with short, smooth-walled, ellipsoidal, monoverticillate Penicillium conidiophores (magnification × 400); **(E)** Microscopic analysis at magnification × 1000; **(F)** Microscopic analysis of colonies grown on Sabouraud’s agar medium plate at 37°C by lactophenol cotton blue staining (magnification × 400); **(G)** Examination of BALF using metagenomic next-generation sequencing (mNGS) revealed TM.

On the 5th day after admission, the patient’s respiratory distress significantly worsened, with a further decrease in percutaneous oxygen saturation and audible crackles in both lungs on auscultation. Despite oxygen mask ventilation with a reservoir bag, oxygen saturation was maintained around 95%. Consequently, the patient was transferred to the intensive care unit (ICU). Treatment in the ICU included oral endotracheal intubation and mechanical ventilation, antimicrobial therapy with meropenem 1.0 q8h plus voriconazole 0.2 q12h, continuous intravenous infusion of norepinephrine to maintain blood pressure, intravenous methylprednisolone sodium succinate 40mg Q12H for anti-inflammatory purposes, fentanyl for pain management, midazolam for sedation, enteral nutrition support, low molecular weight heparin for venous thrombosis prophylaxis, and enhanced sputum suction management. On the second day in the ICU, a bronchoalveolar lavage fluid (BALF) sample was collected for metagenomic next-generation sequencing (mNGS). Two days later (2024-01-12), the NGS results from the BALF showed TM (**Figure 3G**). Consequently, the antifungal regimen was modified, discontinuing voriconazole and meropenem in favor of amphotericin B, gradually titrated up to a therapeutic dose of 0.5mg/kg/day. Additionally, on the 5th day in the ICU (2024-01-15), culture results from the aspirated fluid of the left neck mass also confirmed TM (**Figures 3A, B, D-F**). Subsequent blood culture results from the patient also confirmed TM (**Figure 3C**).

Due to TM being an opportunistic infection and the patient testing HIV-negative, further examination revealed positive AIGA (209.40 AU/mL, detected by chemiluminescence immunoassay, >20 AU/mL considered positive), which remained positive upon retesting (213.00 AU/mL). Consequently, the patient was diagnosed with AOID mediated by AIGA. Treatment continued with intravenous methylprednisolone sodium succinate.

On 2024-01-16, a chest CT scan revealed patchy opacities in both lungs, with partial absorption compared to the scan on 2024-01-11 (**Figures 2D, E**). Additionally, irregular consolidations with cavitation formation were observed in the left subclavian region and left upper mediastinum, accompanied by reduced lymphadenopathy in the hilum and mediastinum compared to previous imaging.

On the 14th day of admission (2024-01-19), the patient’s condition significantly improved with stable circulation, improved lung function, and an oxygenation index of 300, leading to the removal of the endotracheal tube and discontinuation of mechanical ventilation. Concurrently, the widespread rash on the patient’s body markedly improved (**Figure 1B**), and a follow-up examination showed reduced lymphadenopathy (**Figure 2H**).

On the 19th day of admission (2024-01-24), the patient was discharged from the hospital. The treatment was changed from amphotericin B to oral itraconazole capsules, and outpatient follow-up was scheduled with recommendations for regular follow-ups for up to 6 months after discharge.

## 3 Discussion

*Talaromycosis*, a fungal infection, is common in tropical and subtropical parts of Asia. It’s caused by the fungus TM. Each year, approximately 17,300 cases are diagnosed, with about one-third of those cases resulting in death (35). Despite its high mortality rate, this disease has not received much attention globally (35). TM is an opportunistic pathogen, primarily affecting individuals with compromised immune systems, such as those with HIV. Due to the lack of specific clinical manifestations, the disease often presents with atypical symptoms or is masked by other infection symptoms, making diagnosis challenging and leading to a high rate of misdiagnosis.

The patient we reported not only had severe lung infection and acute respiratory failure but also presented with widespread rash, enlarged lymph nodes in multiple areas, mediastinal mass, and cervical vertebral destruction, along with pain behind the sternum, symptoms that could easily lead to a misdiagnosis of lymphoma. We ruled out the possibility of lymphoma through lymph node biopsy, bone marrow cytology, and bone marrow histopathology examinations. Additionally, by conducting mNGS on the pulmonary lavage fluid, we promptly detected the presence of TM. Ultimately, confirmation of TM infection was obtained through fungal culture of the aspirate from the neck mass.

Despite the patient testing negative for HIV, she was infected with TM, raising our suspicions about an underlying condition. It is increasingly recognized that certain conditions can predispose individuals to disseminated infections caused by opportunistic pathogens, including NTM, non-typhoidal Salmonella, *Burkholderia pseudomallei*, *Cryptococcus*, *Histoplasma capsulatum*, and TM (4). Subsequent tests for AIGA were positive twice, confirming our suspicions. The patient was diagnosed with AOID associated with neutralizing AIGA (4).

The detection of antibodies is crucial for diagnosing the disease. However, the median diagnostic time was 12 months, and several cases have reported delays in diagnosis leading to missed treatment opportunities (19). As an emerging infectious disease, timely diagnosis of AOID can be challenging due to limited capabilities at some medical centers. Some authors have suggested using the QuantiFERON Gold In-tube assay to screen for autoantibodies (16). Improving detection methods for AOID is essential to enhance early diagnosis and treatment outcomes. Given the disease’s prevalence in Southeast Asia, clinicians should remain vigilant for AOID in patients from these regions who present with opportunistic infections.

IFN-γ, the sole type II interferon, plays a crucial role in immunity against intracellular bacteria. Genetic susceptibility in its signaling pathway can lead to opportunistic infections, such as those caused by mycobacteria, TM, and Salmonella. Doffinger et al. (6)reported a 47-year-old Filipino male with multiple disseminated infections who had negative results for known Mendelian defects in the interleukin (IL) -12/IFN-γ pathway. While his serum showed an intact response to the purified-protein derivative, it had defective secretion of IFN-γ *in vitro*. They identified a high titer of IgG antibody to IFN-γ, showing it was capable of inhibiting the IFN-γ-dependent augmentation of lipopolysaccharide (LPS)-induced tumor necrosis factor (TNF)-α production (6). Functional studies have shown that the antibody targets a major epitope on free IFN-γ, which is essential for the activation of the IFN-γ receptor (IFN-γR) (36). This interaction inhibits IFN-γ-induced phosphorylation of signal transducer and activator of transcription 1 (STAT1) and subsequent cytokine production (37). Furthermore, the autoantibodies also impair CD4^+^ Th1 and CD8^+^ T cell responses (38). Consequently, patients exhibit impaired intracellular immunity despite having normal lymphocyte counts and cellular responses (2, 6). These studies suggest that in AOID patients, the presence of AIGA is a central pathogenic mechanism, but the development of AIGA remains not fully understood. A study that investigated serial serum samples from a single patient over a 7-year period found that these antibodies appeared 18 months before the clinical onset of symptoms, suggesting their acquisition prior to the disease manifestation (39). Therefore, AIGA is considered pathogenic antibodies rather than antibodies secondary to infection. On the other hand, this disease has been mostly reported in Southeast Asia; even in the USA, most patients (91%) are Asian immigrants, suggesting a genetic propensity for the disease (40). Genetic studies have found a high LD association between several type II HLA alleles with AIGA, including DRB116:02, DRB115:02, DQB105:02, and DQB105:01 (8, 41), and these risk alleles have synergistic effects in contributing to the disease (41). In cases of severe infection, the concentration of IFN should typically increase accordingly. However, in this patient, the serum IFN-γ levels were found to be within the normal range during two separate tests, indicating that some IFN-γ was neutralized by AIGA.

Due to the patient’s confirmed diagnosis of AOID, we believe that solely providing anti-infective treatment may not effectively clear the pathogen, as immune modulation is a crucial part of treatment for such patients. In terms of immunotherapy, the efficacy of corticosteroids, subcutaneous IFN-γ injections, and intravenous immunoglobulin is limited. However, combination therapy with cyclophosphamide and steroids has shown promising results in some patients (42). Rituximab, an anti-CD20 monoclonal antibody, is one of the most extensively studied biologics in patients with adult-onset immunodeficiency diseases. This medication can eliminate circulating B cells, reduce AIGA titers, restore IFN-γ signal transduction, and improve clinical conditions (37). In a Thai study involving 17 patients, 6 of whom received rituximab and 11 received cyclophosphamide due to progressive infections, the effectiveness rates of rituximab and cyclophosphamide were comparable (4/6 and 8/11, respectively) (43). Bortezomib, a proteasome inhibitor targeting plasma cells, has demonstrated additional suppression of autoantibodies following rituximab failure (44). Combining rituximab with bortezomib is likely necessary to prevent generation of new autoantibody-producing plasma cells (45). Daratumumab, an antibody targeting CD38^+^ plasmablasts and plasma cells, has further reduced tissue plasma cells, total IgG levels, AIGA titers, and disease progression (46).

However, when using immunosuppressants or biologics to reduce antibody levels, caution must be exercised to avoid drug-related immunosuppression, which may lead to secondary opportunistic infections. In developing the treatment plan for this patient, we initially recommended rituximab. However, the patient’s family, fully considering the side effects and potential secondary immunosuppression, declined our recommendation. Our multidisciplinary team, including ICU and infectious disease specialists, thoroughly discussed the situation and, taking into account the family’s concerns and the need to avoid drug-related immunosuppression, decided to use corticosteroids alone. This approach aimed to balance reducing antibody titers and avoiding secondary opportunistic infections.

Simultaneously, we implemented a comprehensive treatment plan including amphotericin B, shock management, mechanical ventilation, and nutritional support. Subsequently, the patient’s temperature and inflammatory markers rapidly decreased, and the rash and pulmonary infection significantly improved within a few days. This indicated that the infection was effectively controlled, thereby reinforcing our confidence in the chosen treatment strategy.

## 4 Conclusion

In conclusion, our case highlights the importance of a tailored treatment approach for managing AOID patients, balancing the reduction of pathogenic antibodies with the minimization of secondary infection risks. Although AOID is relatively rare and infrequently encountered in clinical practice, patients with this condition are prone to recurrent and complex infections, such as those caused by TM. Therefore, it is crucial to raise awareness about AOID. For suspected cases, timely antibody screening, early diagnosis, and the use of mNGS to rapidly identify infections can facilitate early treatment and improve patient outcomes.

## Data Availability

The original contributions presented in the study are included in the article/supplementary material. Further inquiries can be directed to the corresponding authors.
